# Scleral resection in chronic central serous chorioretinopathy complicated by exudative retinal detachment

**DOI:** 10.1186/s40662-016-0055-5

**Published:** 2016-09-09

**Authors:** Pradeep Venkatesh, Rohan Chawla, Koushik Tripathy, Harsh Inder Singh, Ravi Bypareddy

**Affiliations:** Department of Ophthalmology, Dr. Rajendra Prasad Centre for Ophthalmology, All India Institute of Medical Sciences, New Delhi, 110029 India

**Keywords:** Nanophthalmos, Uveal effusion syndrome, Diffuse retinal pigment epitheliopathy, Sclerectomy

## Abstract

**Background:**

Effective therapeutic options are limited for the management of chronic central serous chorioretinopathy (CSCR) complicated by exudative retinal detachments (RD). The authors describe the resolution of one such case following partial thickness scleral resection with mitomycin C.

**Case presentation:**

This 39-year-old male presented with a unilateral inferior exudative RD in the right eye. There was no history of steroid use either locally or systemically. The fundus fluorescein angiogram showed window defects and leaks typical of chronic CSCR. The axial length was 21.06 mm in the right eye and 21 mm in the left eye. Thickening of the ocular coats was evident on ocular ultrasound. Considering an axial length in the borderline-low range inferotemporal and inferonasal partial thickness scleral resection with mitomycin C was performed. The exudative RD resolved at 4 months.

**Conclusion:**

Partial thickness scleral resection may be considered as an option for treating chronic CSCR patients with borderline-low axial length complicated by exudative RD.

## Background

Exudative retinal detachment (RD) is a rare complication of chronic central serous chorioretinopathy (CSCR, or diffuse retinal pigment epitheliopathy, DRPE) [1, 2] and is difficult to treat. Suggested etiopathogenesis of CSCR includes increased choroidal vascular permeability, hyperdynamic choroidal circulation, dilation of the Haller’s layer of large choroidal vessels [3], accumulation of fluid in the outer choroid [4], pachychoroid disease [5], and deranged retinal pigment epithelium (RPE) pump mechanism [6].

Available treatment options for chronic CSCR are avoidance of steroids, laser, photodynamic therapy, transpupillary thermotherapy, anti-glucocorticosteroids (oral rifampicin, ketoconazole, mifepristone, finasteride), anti-*Helicobacter pylori* treatment [7], oral acetazolamide, intravitreal anti-vascular endothelial growth factor agents, and aspirin [6].

Idiopathic uveal effusion syndrome (UES) is another important cause of exudative retinal and ciliochoroidal detachment, which may be associated with nanophthalmos. Previously, it was suggested that vortex vein compression by the thick sclera resulted in such detachments in nanophthalmic eyes, which resolved after vortex vein decompression [8]. However, due to the hypoplastic/fragile nature of the vortex veins in such cases, such a procedure could lead to intraoperative amputation or rupture of these veins [9]. Gass JD hypothesized that the hampered permeability of the sclera to protein rich subretinal and suprachoroidal fluid is the cause of UES and that the ‘barrier effect of the sclera is more important than vortex vein obstructing effect’ [9]. He has demonstrated the successful resolution of subretinal fluid in two such eyes within 3 months following sclerectomies and sclerostomies away from the vortex vein [9]. UES with nanophthalmos also benefits from sclerectomy [10] as thick and abnormal sclera is present in such cases. The authors describe the resolution of subretinal fluid in a case of exudative RD in chronic CSCR with borderline-low axial length, following partial thickness scleral resection with mitomycin C (MMC).

MMC has been used successfully for its modulatory effects on wound healing in multiple ocular surgeries including trabeculectomy, pterygium excision, kerato-refractive surgeries, and sclerectomy for retinal detachment in nanophthalmos [11–13]. In scleral resection, MMC is thought to reduce scarring of the sclerectomy bed and prevent recurrence of the exudative retinal detachment due to the regeneration of thick sclera with abnormally arranged collagen fibers [13]. Reported complications of MMC include hypotony secondary to thin cystic bleb following trabeculectomy, endophthalmitis, and corneal or scleral melting [12]. However, we are unaware of any serious adverse effects of the drug when used in conjunction with sclerectomy for nanophthalmos [12, 14].

## Case presentation

**Fig. 1:**
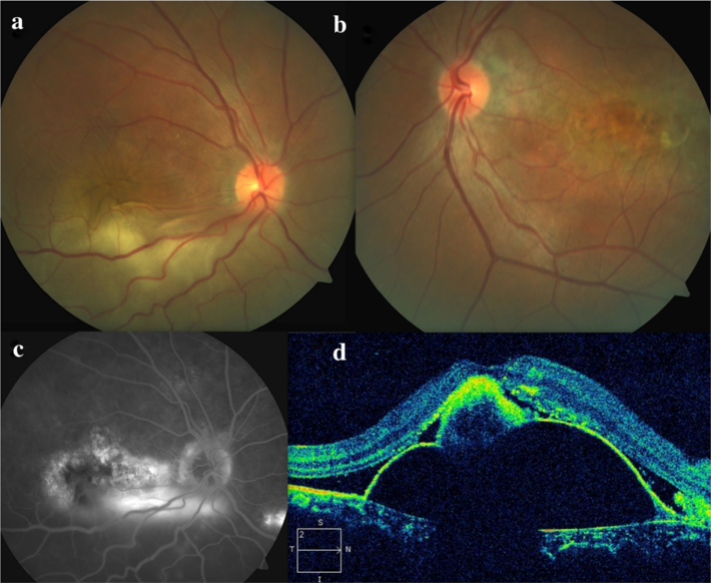
Preoperative fundus findings of the patient. **a** Fundus photograph of the right eye shows inferior exudative retinal detachment with subretinal exudation (fibrin) inferior to the macula. **b** Fundus photograph of the left eye shows pigmentary changes at the posterior pole consistent with diffuse retinal pigment epitheliopathy. **c** Fluorescein angiography (late phase) of the right eye reveals an annular zone of small hyper and hypofluorescent areas corresponding to the mottled appearance of the RPE with pooling of dye in the subretinal space. Most of the hyperfluorescent areas being window defects with few increasing in size and intensity with time suggest active leaks. A small focal leaking area of hyperfluorescence is also seen inferonasal to the disc. **d** Optical coherence tomography (OCT) of the right eye shows the neurosensory detachment at the fovea with some underlying hyperreflective material suggestive of fibrin and a pigment epithelial detachment (PED)

A 39-year-old male presented with painless progressive diminution of vision in the right eye (RE) of 5 years and in the left eye (LE) of 6 months duration. There was no history of chronic drug use, specifically, use of steroid in any form. The visual acuity was 1/60 (RE) and 6/18 (LE). There was no anterior chamber or retrolental cells in both eyes and intraocular pressures were normal. The RE had a bullous inferior RD involving the macula with a smooth surface and shifting fluid. There was whitish subretinal exudation inferior to the macula (fibrin, Fig. 1a). The LE showed pigmentary changes at the posterior pole and multiple pigment epithelial detachments (PEDs) (Fig. 1b). No mass lesion or peripheral break was detected in either eye. Fluorescein angiography (FA) of the RE showed window defects at the posterior pole with leaks at the nasal part of the window defect and inferonasal to the optic disc (Fig. 1c). There was no disc leak or vasculitis. The LE revealed window defects with multiple PEDs. Optical coherence tomography (OCT) of the RE revealed subfoveal fluid with some underlying hyperreflective material suggestive of fibrin and a serous PED (Fig. 1d). OCT of the LE revealed multiple serous PEDs. The sclero-chorioretinal thickness was 2 mm (RE) and 2.01 mm (LE). Axial lengths were 21.06 mm (RE) and 21 mm (LE). Serum cortisol level, blood pressure, and kidney functions tests were normal. A diagnosis of bilateral chronic CSCR with an exudative RD in the RE was made. Some cases of CSR can undergo spontaneous resolution. Thus at the first presentation, the patient was advised to follow-up.

At 1 month, there was no change. Then a 2 month trial of oral rifampicin 300 mg twice daily [15] was tried without success. Laser was not considered as the trans-retinal pigment epithelial leakage was not well defined. Though photodynamic therapy (PDT) [6] has been studied extensively for chronic CSCR [16], its role in chronic CSCR complicated by retinal detachment needs evaluation. Our patient was given the option of PDT, but he declined the therapy due to financial constraints. As the exudative RD was not responding and the eye was small in size, we felt that similar to nanophthalmic eyes, the sclera may be the culprit by not allowing the resolution of the subretinal fluid. Thus, we planned partial thickness scleral resection in the RE as the eye was small with increased thickness of the ocular coats like in nanophthalmos. A proper informed consent for the procedure was obtained. Surgically, an area of 4 mm by 3 mm was marked on the sclera, 4 mm posterior to the insertion of recti in inferotemporal and inferonasal quadrants. Using a blade and crescent knife, a partial thickness (more than 50 %) square of sclera was excised from the marked areas. Mitomycin C 0.02 % was placed on the scleral bed for 2 min after which a thorough wash with the balanced salt solution was performed.

**Fig. 2:**
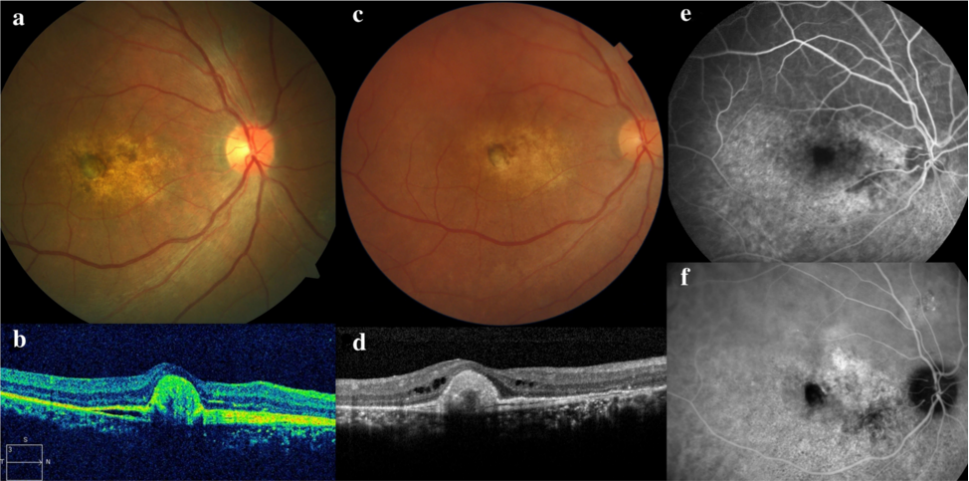
The right eye 4 months (**a**,**b**) and 2 years (**c**,**d**,**e**,**f**) after scleral resection. **a** Four months postoperatively, fundus photograph of the right eye shows total resolution of the exudative detachment with persisting diffuse retinal pigment epitheliopathy at the posterior pole. **b** Four months’ follow-up OCT shows resolution of subretinal fluid and marked reduction in height of the PED with persistence of some of the hyperreflective subretinal fibrin. **c** The right fundus 2 years after surgery, revealing retinal pigment epithelial changes and the absence of subretinal fluid at the posterior pole. **d** OCT of the right eye at 2 years after surgery shows the absence of subretinal fluid in the right eye. **e** The FFA revealed a leopard spot like fluorescence inferiorly. **f** The mid-phase ICGA of the right eye showed a block fluorescence at the foveal center and small pin-point leaks superior to the optic disc

At 2 months, the vision of the right eye had improved to 3/60 and the exudative detachment had started regressing. At 4 months, the vision was 6/60 and the exudative detachment had totally resolved (Fig. 2a). OCT revealed resolution of subretinal fluid and marked reduction in the height of the PED with persistence of some of the hyperreflective subretinal fibrin (Fig. 2b).

After 2 years of surgery, the right eye had achieved a best-corrected visual acuity of 6/36. The right eye had attached retina with a focal elevated pigmented area at the fovea surrounded by hypopigmentation (Fig. 2c). OCT showed the absence of subretinal fluid. Few intraretinal cystoid spaces and a subretinal mound presumably due to fibrin were visible (Fig. 2d). The FFA revealed the absence of any active leak and leopard spot like fluorescence in the inferior retina reminiscent of previous retinal detachment (Fig. 2e). The mid-phase indocyanine green angiogram (ICGA) of the right eye was suggestive of DRPE. The foveal center appeared dark, likely due to blocked fluorescence by the focal retinal pigment epithelial hyperplasia as seen on the fundus image. Small pinpoint leaks were visible superior to the disc (Fig. 2f).

**Fig. 3:**
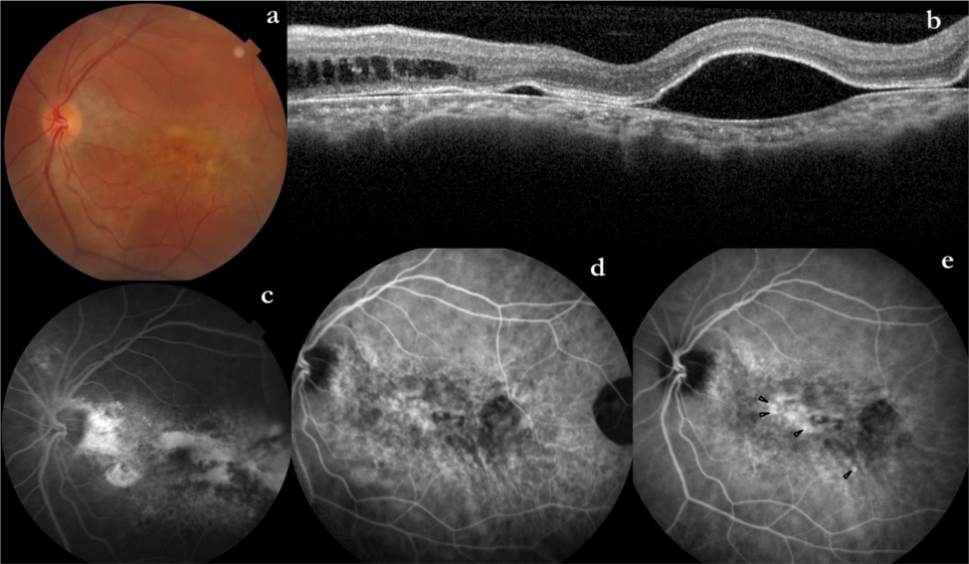
Evaluation of the left eye at 2 years’ follow-up. **a** The fundus of the left eye at 2-years follow-up. **b** The OCT of the left eye showed multiple PEDs. **c** The FFA of the left eye revealed mottled hyper- and hypofluorescence, and no definite focal leak was evident. **d** Early phase ICGA of the left eye showed inferiorly enlarged choroidal vessels and granular fluorescence at the posterior pole. **e** Mid-phase ICGA revealed increased choroidal permeability and few active pin point leaks (black arrowhead)

The left eye had retained 6/18 vision and showed RPE changes and subretinal yellowish deposits (Fig. 3a). The OCT showed PEDs temporal to the fovea; and nasal intraretinal cystoid spaces (Fig. 3b). The FFA of the left eye revealed areas of mottled hyper and hypofluorescence with broad areas of staining, but no definitive focal leak could be identified (Fig. 3c). ICGA (early phase) of the left eye showed patchy granular fluorescence at the posterior pole, and dilated large choroidal vessels inferior to the fovea (Fig. 3d). Diffusely increased choroidal hyperfluorescence around this region at the posterior pole persisting in the late phase, suggestive of increased choroidal permeability was also seen. In the mid phase, few active pin point leaks were also evident (black arrowhead, Fig. 3e).

## Conclusions

Causes of exudative RD include hypertension [17], Vogt-Koyanagi-Harada syndrome, posterior scleritis, metastasis, uveal effusion syndrome, nanophthalmos and other ocular tumors. In our patient, blood pressure was normal and there was no evidence of intraocular inflammation. Our patient did not have pain and subtenon fluid on ultrasound typical of posterior scleritis. Ocular tumor or metastasis was ruled out by indirect ophthalmoscopy and ocular ultrasound.

Uveal effusion syndrome was ruled out as there was no ciliochoroidal detachment. The most important differentials in our case included chronic CSCR [1] and serous RD secondary to nanophthalmos [18]. Considering the clinical picture, fluorescein angiography and OCT findings, our primary diagnosis was chronic CSCR. The eye of our patient was not nanophthalmic by strict diagnostic criterion (axial length < 20 mm) [19], but it was still a small eye with borderline axial length. The thickness of the ocular coats was also increased. Thus, we hypothesized that additionally in our patient there may be a component of reduced transscleral outflow that is seen in nanophthalmic eyes. This reduced outflow was probably unable to balance the marked exudative inflow due to chronic CSCR and resulted in a persistent exudative RD.

We have previously demonstrated the benefit of partial thickness scleral resection with mitomycin C in nanophthalmic eyes with exudative RD [12]. A similar procedure was carried out in our patient. However, chronic CSCR with exudative RD may also resolve spontaneously [2]. Yet a slow but dramatic resolution of the subretinal fluid and improvement of visual acuity of our patient after this particular intervention suggests that the surgery was contributing to the resolution of the detachment. We hypothesize that smaller eyes with compromised transscleral outflow may be at higher risk of developing large exudative RDs in the presence of other pathologies like chronic CSCR. These eyes may benefit from partial thickness scleral resection surgery to improve transscleral outflow. The limitation of our hypothesis is that it is based upon a single case report and we do not have long-term results after the surgery. Larger case series with similar ocular parameters and longer follow-up are required to further elaborate the role of such procedures.

## Abbreviations

CSCR: Central serous chorioretinopathy
DRPE: Diffuse retinal pigment epitheliopathy
ICGA: Indocyanine green angiogram
LE: Left eye
MMC: Mitomycin C
OCT: Optical coherence tomography
PDT: Photodynamic therapy
PED: Pigment epithelial detachments
RD: Retinal detachment
RE: Right eye
RPE: Retinal pigment epithelium
UES: Uveal effusion syndrome
